# The Enigma of *NTH2* Gene in Yeasts

**DOI:** 10.3390/microorganisms12061232

**Published:** 2024-06-19

**Authors:** Sergi Maicas, Ruth Sánchez-Fresneda, Francisco Solano, Juan-Carlos Argüelles

**Affiliations:** 1 Departament de Microbiologia i Ecologia, Facultat de Ciències Biològiques, Universitat de València, 46100 Burjassot, Spain; 2 Área de Microbiología, Facultad de Biología, Universidad de Murcia, 30100 Murcia, Spain; ruth.sanchez1@um.es; 3 Departamento de Bioquímica y Biología Molecular B e Inmunología, Facultad de Medicina Campus de Ciencias de la Salud, Universidad de Murcia, 30120 Murcia, Spain; psolano@um.es

**Keywords:** *NTH2* gene, neutral trehalase, Nth1/Nth2 enzymes, trehalose, yeast

## Abstract

The enzymatic hydrolysis of the non-reducing disaccharide trehalose in yeasts is carried out by trehalase, a highly specific α–glucosidase. Two types of such trehalase activity are present in yeasts, and are referred to as neutral and acid enzymes. They are encoded by distinct genes (*NTH1* and *ATH1*, respectively) and exhibit strong differences in their biochemical and physiological properties as well as different subcellular location and regulatory mechanisms. Whereas a single gene *ATH1* codes for acid trehalase, the genome of some yeasts appears to predict the existence of a second redundant neutral trehalase, encoded by the *NTH2* gene, a paralog of *NTH1*. In *S. cerevisiae* the corresponding two proteins share 77% amino acid identity, leading to the suggestion that *NTH2* codes for a functional trehalase activity. However, Nth2p lacks any measurable neutral trehalase activity and disruption of *NTH2* gene has no effect on this activity compared to a parental strain. Likewise, single nth1Δ and double nth1Δ/nth2Δ null mutants display no detectable neutral activity. Furthermore, disruption of *NTH2* does not cause any apparent phenotype apart from a slight involvement in thermotolerance. To date, no evidence of a duplicated NTH gene has been recorded in other archetypical yeasts, like *C. albicans* or *C. parapsilosis*, and a possible regulatory mechanism of Nth2p remains unknown. Therefore, although genomic analysis points to the existence, in some yeasts, of two distinct genes encoding trehalase activities, the large body of biochemical and physiological evidence gathered from *NTH2* gene does not support this proposal. Indeed, much more experimental evidence would be necessary to firmly validate this hypothesis.

## 1. Introduction

Trehalose is a widely conserved and physiologically relevant non-reducing disaccharide which consists of two glucose molecules linked by an α(1,1) bond. This sugar plays a crucial role in various biological processes that are essential for maintaining cellular homeostasis in numerous organisms [1]. The importance of trehalose is underscored by its involvement in cell protection against both nutritional and environmental stress conditions (i.e., starvation, dehydration, heat or freezing). This success can largely be explained by its chemical structure and singular physical properties, as it is the thermodynamically and kinetically most stable disaccharide in the biosphere [1,2,3]. Furthermore, recent intensive research also suggests promising applications of trehalose in food, cosmetic or clinical therapy [1]. Notably, trehalose is undergoing intensive investigation for its relevant neuroprotective effects, particularly when it is used to treat devastating neurodegenerative diseases like Huntington’s, Alzheimer’s or Parkinson’s disease [1]. Indeed, trehalose is able to cross the blood–brain barrier and may induce direct protection of proteins prone to forming aggregates, and it could be involved in the activation of autophagy [1]. In most organisms, hydrolysis of trehalose is carried out by enzymes known as trehalases (E.C. 3.2.1.28). These are a kind of α-glucosidase rigorously specific to trehalose as a sole substrate, which is cleaved off in two molecules of glucose [3,4,5]. Several prokaryotes and eukaryotes display two mechanistic exceptions to this rule: (i) trehalose phosphorylase, which catalyzes the phosphorolysis of trehalose to produce glucose-1-phosphate and glucose, and (ii) phosphotrehalose that converts the intermediate trehalose-6-phosphate into glucose and glucose-6-phosphate [3,4,5].

Using yeasts as model organisms, an important distinction can be envisaged between two key physiological processes that require trehalose hydrolysis. The first course is the rapid mobilization of metabolic-responsive endogenous trehalose, which serves as an internal energy reserve that is crucial to the cell’s rapid response to sudden demands. This form of trehalose serves as an internal energy reserve that is crucial to the cell’s rapid response to metabolic demands, particularly under stress conditions like nutrient scarcity or environmental stress. The second process is the utilization of exogenous free trehalose, which may be available in the yeast’s environment. After hydrolysis, the released glucose can be imported into the cells and further used either as an energy source or for other physiological activities. This external trehalose can be imported into the cell and used as an energy source or for other metabolic functions. These two processes are facilitated by two distinct trehalases, each encoded by different, unrelated genes. These trehalases exhibit significant biochemical and genetic differences. One of the key differences lies in their subcellular locations: the trehalase responsible for mobilizing endogenous trehalose is typically found in the cytosol, whereas the trehalase involved in utilizing exogenous trehalose is often associated with the cell membrane or other compartments. Moreover, these trehalases differ in their catalytic parameters. The cytosolic trehalase generally has a higher affinity for trehalose and is regulated in a way that allows a rapid response to internal metabolic cues. In contrast, the membrane-associated trehalase may have different kinetic properties, which are optimized for processing external sources of trehalose that are less consistently available. Regulatory controls also vary between these trehalases. The endogenous trehalase is tightly regulated by intracellular signals that reflect the cell’s metabolic state, ensuring that trehalose is mobilized when the cell requires energy. On the other hand, the exogenous trehalase is regulated in a manner that aligns with the availability of trehalose in the environment, allowing the cell to take advantage of external nutrient sources when present. This differentiation between trehalase functions highlights the complex regulatory networks that enable yeast cells to efficiently manage their energy resources and adapt to fluctuating environmental conditions. Understanding these mechanisms not only provides insights into yeast metabolism but also offers broader implications for the study of energy regulation and stress responses in other organisms [3,5,6]. They only share a strict specificity for trehalose as a substrate and are usually distinguished as cytosolic or neutral trehalases (Nth1p/Nct1p), which are engaged in the mobilization of endogenous trehalose, which is activated by some divalent cations (Mg^2+^ and Ca^2+^) and regulated by reversible phosphorylation mediated by cAMP-dependent protein kinases. In contrast, the so-called acid trehalases (Ath1p/Atc1p) are cell-linked external or vacuolar enzymes that are responsible for cell growth on exogenous trehalose and subjected to glucose repression [3,4,5].

It should be mentioned, however, that in some yeasts that are considered important for biotechnological processes, such as *Saccharomyces cerevisiae*, which is used in the production of bioethanol and other fermentation products, and in yeasts relevant as human pathogens, such as *Candida albicans*, which can cause opportunistic infections, a second gene known as *NTH2* has been described. This gene codes for a putative neutral trehalase.

This marked distinction between the functions of trehalase–trehalose systems in fungi highlights the complex regulatory networks that enable fungal cells to efficiently manage their energy resources and adapt to fluctuating environmental conditions. A precise understanding of the mechanisms involved would not only provide new insights into fungal metabolism, but would also have broader implications for the study of energy regulation and stress-response circuits in other organisms [1,3,4,5]. In this way, the main features and putative relationships between both trehalases have been exhaustively revised and will not be analyzed here [3,4,5]. It should be mentioned, however, that in some yeasts that are considered important for biotechnological processes (i.e., *Saccharomyces cerevisiae* utilized in the production of bioethanol and other fermentation products) as well as in yeasts that are relevant as human pathogens (i.e., *Candida albicans* and other “non-albicans” species of *Candida* that cause both superficial and systemic opportunistic infections) a second gene (*NTH2*) has been described, coding for a putative neutral trehalase. Notably, despite its identification, the main features and biological roles of which remain elusive [2,7].

## 2. Does the *NTH2* Gene Code for a Functional Neutral Trehalase in Yeasts?

**Figure 1:**
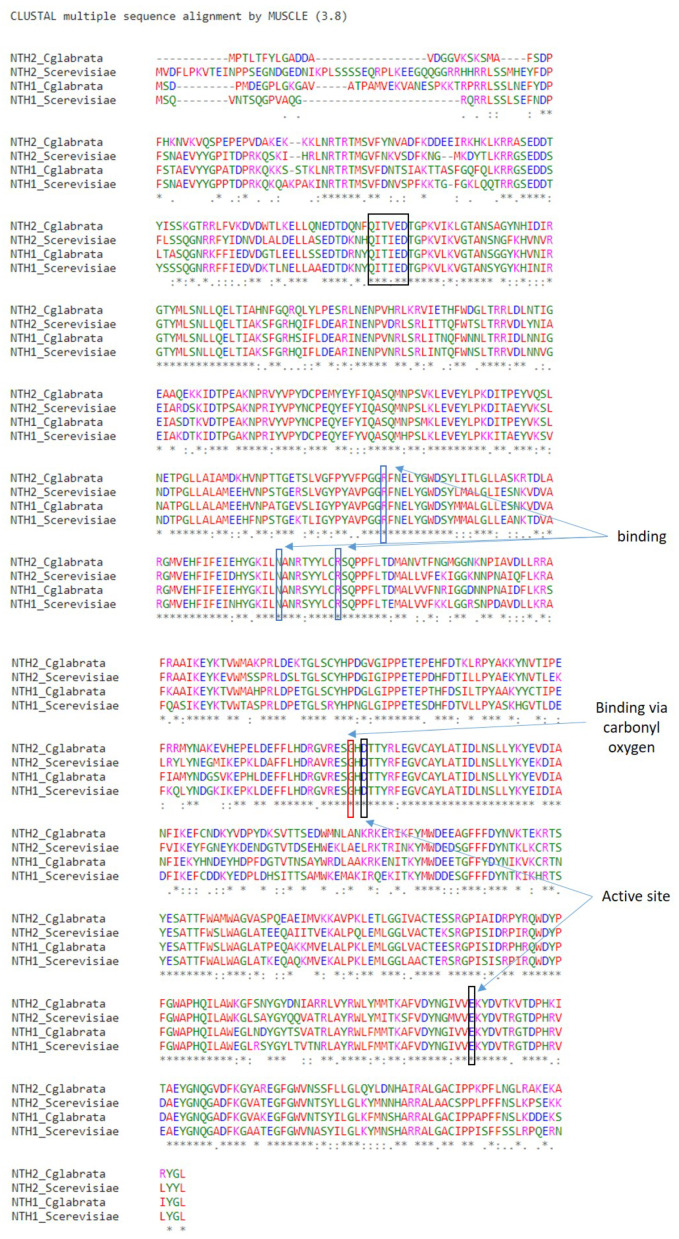
Alignment of the amino acid sequences corresponding to Nth1 and Nth2 enzymes in the yeasts *Candida glabrata* and *Saccharomyces cerevisiae*. The alignment was performed using the MUSCLE program (Multiple Sequence Comparison by Log-Expectation). The text indicates the residues that are identical (*), conserved substitutions residues (:) and semi-conserved substitutions (.). Boxes indicate the essential residues involved in trehalose binding and catalytic activities.

Unlike acid trehalases, where a unique gene (*ATH1*) codes for acid trehalase, a search in the genome databases predicts the existence of a redundant neutral trehalase activity in several yeasts, encoded by the *NTH2* gene, a paralog of *NTH1* [2,7,8]. In *Saccharomyces cerevisiae* the corresponding proteins appear to share 77% identity. While it has been proposed that Nth2p is a true functional trehalase that is directly involved in the mobilization of stored endogenous trehalose [2,7,8], we are inclined to believe that the available data are inconsistent with this statement. Indeed, much more experimental evidence would be necessary to firmly establish the above-indicated hypothesis. The following arguments support this reluctant view: Apart from in *S. cerevisiae*, the presence of two neutral trehalase activities has only been extensively demonstrated in the yeast *Candida glabrata* (Nth1p consists of 769 amino acids/87.4 kDa, whereas Nth2p contains 750 amino acids/86.5 kDa) [7]. This coincidence reinforces the suggestion that *C. glabrata* fits better into Saccharomycetaceae clade than in the *Candida* clade [9]. Notably, in *S. cerevisiae* the Nth2p enzyme shows a higher resemblance with its own Nth1p than with the ortholog Nth2p present in *C. glabrata* (Figure 1) Furthermore, the highest degree of homology (71% amino acids identity) corresponds to neutral trehalases from *C. glabrata* [7], while an elevated similarity also exists between the two Nth1 enzymes from these two yeasts (Figure 1). Furthermore, a comparison of sequences between *NTH1* and *NTH2* genes confirms they do not contain a transmembrane domain or a signal sequence (Figure 1) [3,5]. In addition, a careful database search (Swiss Prot) points to the presence of *NTH2*-related genes in the phytopathogenic filamentous fungi *Eremothecium gossypii* and in the lager yeast *Saccharomyces pasterianus*, which have not been characterized yet. Nevertheless, thus far, no evidence concerning this duplicated *NTH2* gene has been found in other relevant yeasts. Thus, in the opportunistic pathogen *C. albicans*, only one gene appears to code for neutral trehalase, although there is still a need for further research [10]. Remarkably, the homozygous deletion of the single *ATH1* and *NTH1* genes completely abolishes trehalase activity in *C. parapsilosis* [11,12]. More intriguing is the observation that after precise catalytic measurements, the Nth2p has no neutral trehalase activity in *S. cerevisiae* and disruption of *NTH2* gene does not modify this activity compared with a parental strain [2]. Likewise, single nth1Δ and double nth1Δ/nth2Δ null mutants show no detectable neutral activity [7,9]. It might be expected that, if *NTH2* encodes a genuine functional neutral trehalase, it would compensate for the lack of Nth1p activity. In contrast, although overexpression of *NTH2* in a nth1Δ mutant induces a certain increase in trehalose hydrolysis, the result is unclear, since acid trehalase may be operative in these cells [8,12]. Another important clue is the absence of a specific associated phenotype to disrupt *NTH2*. Thus, in *S. cerevisiae* an intact *NTH2* gene appears to be required for cell recovery upon heat shock and saline stress [7,13]. As a rule, the phenotypes displayed by heterozygous nth1Δ/Nth2 and nth1Δ/nth2Δ null mutants appear quite similar, with a slight difference concerning the capacity of trehalose accumulation [8,14]. In turn, the role of Nth2p in *C. glabrata* is a mystery. In tests on virulence factors involved in gut colonization, *NTH2* gene is upregulated inside macrophages and its deletion reduces cell survival, but it does not play a conspicuous role in this process [7], although in many pathogenic fungi, stored trehalose is an element of virulence [13,15].

## 3. Hypothetical Roles of *NTH2* Genes in the Physiology of Yeasts

The information currently available does not allow the formulation of a convincing proposition concerning a main function of *NTH2* gene in the physiology of yeasts. Some preliminary data suggest that it might have an important role in the fine-tuning of trehalose metabolism, possible by providing an additional level of regulatory control under specific nutritional and/or environmental conditions [2,4,8]. For instance, Nth2p might be involved in trehalose hydrolysis when yeast cells experience a certain type of abiotic stress or when there is a sudden need for energy that cannot be satisfied by the primary trehalase alone. Furthermore, the potential significance of *NTH2* in biotechnological applications and pathogenicity adds an intriguing dimension to its extensive research. Thus, in many industrial yeast strains, a clear elucidation of the *NTH2* function could lead to enhanced metabolic engineering strategies, improving the yield of bioethanol or other valuable bioproducts by optimizing the endogenous trehalose content. In turn, in pathogenic yeasts, this knowledge would provide new insight regarding the adaptative processes of these organisms to the host body during a productive infection, revealing new potentially interesting targets for antifungal therapies [1,2,3,5,8].

## 4. Conclusions and Perspectives

We conclude that although the sequence data seem to suggest that *NTH2* gene might code for a neutral trehalase in yeasts, extensive and precise genetic and biochemical analysis do not support this proposal. Nth2p lacks any measurable trehalase activity, while disruption of *NTH2* gene has no effect on both the endogenous trehalose content and its mobilization recorded in wild type cells. Furthermore, the *nth2*Δ null mutants do not display an evident phenotype, beyond a certain involvement in tolerance to heat shock and saline stress, but these observations require further confirmation. Other questions related to catalytic properties, regulatory mechanisms and physiological roles played by this putative neutral trehalase should also be readdressed. Further investigations should include gene knockout studies and in-depth biochemical characterization of Nth2p enzyme and the analysis of its expression patterns displayed by the distinct trehalases under various growth conditions. Insights gained from *NTH2* research could promote the development of more robust industrial yeast strains or the obtention of novel more potent and safer antifungals, providing stronger support for the interconnection between basic research and applied sciences. Therefore, although *NTH2* gene must play a physiological role in yeasts under still-unknown conditions, to date, it remains an enigma.
